# YOD1 stabilizes HIV-1 Nef via K6-linked deubiquitination to promote viral infectivity and impair immune recovery

**DOI:** 10.3389/fimmu.2026.1936616

**Published:** 2026-10-23

**Authors:** Jin Liu, Qian Xu, Yu Jiang, Zhouzhou Zhang, Guiyan Li, Minyan Yao, Tiantian Gan, Yiming Zheng, Yujuan Yan, Yueping Zhu, Saihong Gu, Ming Li, Li Zhu, Jianping Zhang, Chuanwu Zhu, Feng Qian

**Affiliations:** 1 Department of Infectious Diseases, The Affiliated Infectious Diseases Hospital, Suzhou Medical College of Soochow University, The Fifth People’s Hospital of Suzhou, Suzhou, China; 2 Department of Infectious Diseases, Zhangjiagang Hospital Affiliated to Soochow University, Suzhou, China; 3 Department of Pharmacy, The Affiliated Infectious Diseases Hospital, Suzhou Medical College of Soochow University, The Fifth People’s Hospital of Suzhou, Suzhou, China; 4 Department of Urology Surgery, The Affiliated Suzhou Hospital of Nanjing Medical University, Suzhou Municipal Hospital, Gusu School, Nanjing Medical University, Suzhou, China; 5 Department of Tuberculosis, The Affiliated Infectious Diseases Hospital, Suzhou Medical College of Soochow University, The Fifth People’s Hospital of Suzhou, Suzhou, China

**Keywords:** HIV-1, immune reconfiguration, Nef, vitamin C, YOD1

## Abstract

**Background:**

Immune non-response to antiretroviral therapy remains a major challenge in HIV-1 management, yet the underlying molecular mechanisms are incompletely understood.

**Methods:**

In this study, we performed comparative quantitative proteomics on samples from immunonon-responsive (INR) and immune-responsive (IR) individuals, identifying the deubiquitinase YOD1 as one of the most significantly upregulated proteins in INR patients, accompanied by hyperactivation of ubiquitin‑proteasome pathways.

**Results:**

Through immunoprecipitation coupled with mass spectrometry, we found that YOD1 associates with the HIV-1 Nef protein, a key viral factor that subverts host immunity. Mechanistically, YOD1 specifically removes K6‑linked polyubiquitin chains from Nef via its catalytic cysteine residue (C155S), thereby preventing proteasomal degradation and dose‑dependently stabilizing Nef protein levels without affecting its transcription. This regulation was selective for Nef, as YOD1 did not alter other HIV‑1 proteins (gp120, Vif, or Tat). In HIV‑1‑infected THP‑1 macrophages and clinical PBMCs from people living with HIV, YOD1 expression correlated positively with Nef levels and viral load; knockdown or catalytic inactivation of YOD1 markedly increased Nef ubiquitination, reduced p24 antigen secretion, and suppressed virus production. Importantly, screening of nutritional compounds revealed that Vitamin C (VC) promotes YOD1 degradation via the ubiquitin‑proteasome pathway, leading to downregulation of Nef, inhibition of HIV‑1 replication, and restoration of CD4+ T‑cell counts. These antiviral and immune‑restorative effects of VC were completely abrogated upon YOD1 depletion, confirming their dependency on YOD1 targeting.

**Conclusion:**

Collectively, our findings establish YOD1 as a critical host factor that sustains HIV‑1 Nef stability and viral infectivity through K6‑linked deubiquitination, and they identify Vitamin C as a clinically accessible agent that degrades YOD1, offering a potential adjuvant strategy to improve immune recovery in non‑responding patients.

## 1 Introduction

Human immunodeficiency virus (HIV) primarily targets CD4^+^ T cells. A defining characteristic of people living with HIV (PLWH) is the irreversible impairment of the number, diversity, and function of CD4^+^ T cells within the body (1). Consequently, achieving optimal virological suppression-defined as plasma viral RNA levels below the detection limit and maintaining quality of life among PLWH depend on highly active antiretroviral therapy (HAART) (2). This therapy effectively suppresses viral replication and, in many patients, increases CD4^+^ T cell counts and partially restores immune function; however, residual immune activation and incomplete recovery remain significant challenges (3). For infected individuals whose CD4^+^ T cell counts recover to 500 cells/μL, immune reconstitution is considered favorable, significantly reducing the risk of opportunistic infections (4). However, immune reconstitution is not always achieved, and substantial interindividual variation in CD4^+^ T cell recovery has been reported (5). Previous studies have documented that despite long-term HAART and effective viral suppression, approximately 10-40% of infected individuals failed to achieve satisfactory CD4^+^ T cell recovery and remained severely immunocompromised (6). This insufficient immune recovery has been associated with AIDS-related and non-AIDS-related complications, including metabolic disorders, renal impairment, opportunistic infections, and malignancies, ultimately increasing mortality. This population is referred to as immunologically non-responsive individuals (or immunological non-responders), characterized by incomplete immune reconstitution (7).

At present, considerable controversy exists worldwide regarding the definition of “poor immunological responders”. One definition classifies individuals with CD4^+^ T cell counts persistently below 200 cells/μL after 4–7 years of continuous antiretroviral therapy as “poor immunological responders” (8). According to the World Health Organization, patients with an increase of less than 100 CD4^+^ T cells/μL from baseline after two years of effective treatment are defined as poor immunological responders (9). In contrast, a CD4^+^ T cell count exceeding 500 cells/μL is considered indicative of successful immune reconstitution (10). Overall, failure to achieve normal CD4^+^ T cell counts after treatment is regarded as incomplete immune reconstitution. Rita Rb-Silva and colleagues, through a systematic review of multiple original studies, proposed that immunological non-responders (INRs) are defined by an absolute CD4^+^ T cell count below 350 cells/μL after 24 months of antiretroviral therapy (11). This definition helps identify INRs whose CD4^+^ T cell counts should have reached maximal recovery but remain below expected levels. Incomplete immune reconstitution is currently attributed to the complex interplay of multiple mechanisms. These mechanisms may involve impaired function of lymphoid organs and the thymus, persistent replication within HIV reservoirs, heightened activation mediated by CCR5, co-infections, microbial translocation across the intestinal barrier, and CD4^+^ T cell depletion (12). Among these factors, CD4^+^ T cell destruction following HIV infection and immune hyperactivation driven by viral reservoirs play major roles in incomplete immune reconstitution.

To evade host antiviral defenses and exploit host cells for replication, the HIV-1 genome encodes three structural proteins (Gag, Pol, and Env), two regulatory proteins (Tat and Rev), and several accessory proteins (Nef, Vif, Vpr, and Vpu) (13). Each viral protein performs distinct functions according to its specific role in the viral life cycle. *In vivo*, these accessory proteins create an intracellular environment conducive to viral replication, promote viral transmission, and enhance pathogenicity (14). Among these proteins, Nef (Negative Regulatory Factor) is a highly conserved early gene product encoded by the genomes of HIV-1, HIV-2, and simian immunodeficiency virus (SIV). It is also the most immunogenic among all HIV-1 accessory proteins (15). Nef plays a crucial role in HIV-1 pathogenesis, disease progression, and immune reconstitution.

The failure of CD4^+^ T cell counts to return to normal levels primarily results from insufficient production and excessive depletion involving multiple mechanisms. Nef has long been recognized for its ability to lower the activation threshold of resting CD4^+^ T cells, promote cellular activation, and facilitate HIV-1 infection. It also contributes to the establishment and maintenance of viral reservoirs during HIV-1 latency (16, 17). Nef downregulates HLA-A/B, SERINC5 (serine incorporator 5), and the CD3-TCR (T cell receptor) complex, thereby enhancing viral infection, replication, and immune evasion (18, 19). Nef has been reported to stimulate the immune system through lipid rafts, facilitating reservoir-associated viral reactivation and inducing CD4^+^ T cell apoptosis. This immune stimulation is mediated by the lipid raft pathway (20). Recent studies have suggested that combining Nef inhibitors with latency-reversing agents (LRAs) and therapeutic vaccines may reduce or even eliminate HIV reservoirs (21).

The ubiquitin-proteasome system (UPS) plays a critical role in diverse cellular processes, including protein degradation, molecular interactions, and signal transduction. During the viral life cycle, ubiquitination is closely linked to HIV-1 transcription, latency, reverse transcription, and antiviral restriction. Database analyses indicate that approximately 17% of the 103 identified deubiquitinating enzymes (DUBs) interact functionally with HIV-1. In terms of E3 ubiquitin ligases, 95 ligases-approximately 25%-have been reported to be associated with HIV-1 (22). The viral infectivity factor (Vif) induces the polyubiquitination and subsequent degradation of APOBEC3G (apolipoprotein B mRNA editing enzyme catalytic polypeptide-like 3G) (23). The E3 ubiquitin ligase MDM2 participates in both Vif degradation and enhancement of Tat transcriptional activity. A 2017 study revealed that the E3 ubiquitin ligase Praja-2 (PJA2) ubiquitinates Tat in a non-degradative manner (24). Beyond transcription, ubiquitination is also involved in viral particle assembly and release. The E3 ubiquitin ligase NEDD4-like protein (NEDD4L) interacts with Gag to promote the release of HIV-1 particles (25). The E3 ubiquitin ligase MARCH8 restricts viral particle formation by downregulating multiple host transmembrane proteins (26). Regarding DUBs involved in HIV-1 replication, research has progressively explored both broad-spectrum DUB inhibitors (DUBi) and small-molecule inhibitors targeting specific DUBs. Specific inhibitors P22077 and PR-619, which target USP7 and USP47 respectively, disrupt Gag processing and reduce the infectivity of released HIV-1 particles (27). However, the identification and regulatory mechanisms of DUBs associated with the HIV-1 accessory protein Nef remain largely unexplored.

In this study, we provide compelling evidence that the deubiquitinating enzyme YOD1 directly associates with the HIV-1 Nef protein. Through quantitative proteomic profiling, we identified YOD1 as one of the most significantly upregulated proteins in INR patients, correlating with aberrant activation of ubiquitin-proteasome pathways. Mechanistically, YOD1 selectively removes K6-linked polyubiquitin chains from Nef via its catalytic cysteine residue (C155S), thereby shielding Nef from proteasomal degradation and stabilizing its protein levels in a dose-dependent manner, without affecting Nef mRNA transcription. This regulation is selective for Nef, as YOD1 does not alter other HIV-1-encoded proteins. In HIV-1-infected macrophages and patient-derived PBMCs, YOD1 expression positively correlates with viral load and Nef levels; depletion or catalytic inactivation of YOD1 markedly enhances Nef ubiquitination, reduces p24 antigen secretion, and suppresses virus production. Notably, we found that Vitamin C promotes YOD1 degradation via the ubiquitin-proteasome pathway, thereby destabilizing Nef, inhibiting HIV-1 replication, and restoring CD4^+^ T cell counts—effects entirely dependent on YOD1 targeting. Collectively, our findings establish YOD1 as a critical host factor that sustains Nef stability and viral infectivity through K6-linked deubiquitination, and identify Vitamin C as a clinically accessible agent that degrades YOD1, offering a potential adjuvant strategy to improve immune recovery in immunological non-responders.

## 2 Materials and methods

### 2.1 Cell culture and reagents

HEK293T, HeLa, and THP-1 cell lines were obtained from the American Type Culture Collection (ATCC). HEK293T and HeLa cells were cultured in Dulbecco’s Modified Eagle Medium (DMEM; HyClone) at 37 °C in a humidified atmosphere containing 5% CO_2_. THP-1 cells were cultured in RPMI 1640 medium (HyClone) at 37 °C in 5% CO_2_, supplemented with 10% fetal bovine serum (FBS; Gibco, Life Technologies), 100 U/mL penicillin, and 100 μg/mL streptomycin. Human PBMCs were from The Fifth People’s Hospital of Suzhou. Transient transfections were performed using LongTrans (UcallM, TF/07) according to the manufacturer’s instructions.

### 2.2 Plasmids and reagents

Plasmids encoding HIV-1 viral proteins, including Flag-tagged Nef (Flag-Nef), Vif (Flag-Vif), gp120 (Flag-gp120), Tat (Flag-Tat), and the HIV-1 molecular clone NL4-3, were generously supplied by Dr. Binlian Sun from Jianghan University, China. The Myc-His-YOD1 (MH-YOD1/Myc-YOD1) expression plasmid was sourced from Unibio. Flag-HA-YOD1(FH-YOD1), HA-Ub and its lysine-mutant derivatives (HA-R6K, HA-R11K, HA-R27K, HA-R29K, HA-R33K, HA-R48K, and HA-R63K), in which all lysine residues in the ubiquitin gene were replaced with arginine except for the specified lysine, were kindly provided by Hui Zheng from Soochow University, China. All guide RNAs (gRNAs) were procured from WZ Biosciences Inc. YOD1-specific siRNA and negative control siRNA were purchased from Biomics Inc. (China).

### 2.3 RNA isolation and RT-qPCR

The extraction of total RNA from cells was performed using TRIzol reagent (Invitrogen, USA), and 600 ng of this RNA was then used to create complementary DNA (cDNA) with the 5× All-In-One RT Master Mix (Abcam, Cat. No. G490). Afterward, RT-qPCR was employed to analyze mRNA expression levels, utilizing 2×SYBR Green qPCR Master Mix (Selleck, Cat. No. B21202). The sequences of the primers utilized were as follows:

HIV-1 Nef: forward primer, 5′-GCCTGGCTAGAAGCACAAGAA-3′; reverse primer, 5′-GTGATGAAATGCTAGGCGGC-3′.

YOD1: forward primer, 5′-CAATGGGGATACCATTCTGG-3′; reverse primer, 5′-ACCACGGTTCTGGTAAGC-3′.

*β*-actin: forward primer, 5′-ACCAACTGGGACGACATGGAGAAA-3′; reverse primer, 5′-ATAGCACAGCCTGGATAGCAACG-3′.

Target gene mRNA expression levels were standardized to *β*-actin and assessed from three independent trials. Results are expressed as the mean along with the standard deviation (SD).

### 2.4 Immunoblotting and immunoprecipitation

Cells were lysed using a lysis buffer containing 1% NP-40, 150 mM NaCl, 0.5 mM EDTA, and 20 mM Tris-HCl at pH 7.4. Prior to lysis, phenylmethylsulfonyl fluoride (PMSF) at a concentration of 50 μg/mL and a protease inhibitor cocktail (Sigma, USA) were added to the buffer. The cell lysates were subjected to centrifugation at 12,000 × g for 15 minutes at 4 °C. Protein levels were measured with a microplate reader, and the same quantities of lysate were utilized for Western blotting and immunoprecipitation.

For immunoprecipitation, specific antibodies were introduced to the lysates and incubated overnight at 4 °C with continuous rotation. Subsequently, Protein G agarose beads (Millipore, Cat. No. 16-266) were added and incubated for an additional 4 hours at 4 °C with rotation. The resulting immunocomplexes were washed five times with the lysis buffer. For ubiquitination assays, cells were lysed in a 1% NP-40 buffer supplemented with 10 mM N-ethylmaleimide (NEM). The immunocomplexes were washed once with a high-salt buffer (500 mM NaCl) and four times with a normal buffer (150 mM NaCl). The bound proteins were eluted by incubating them in SDS loading buffer containing *β*-mercaptoethanol for 10 minutes. Proteins were resolved using SDS-PAGE and subsequently transferred onto PVDF membranes (Millipore). The membranes were blocked with 5% nonfat milk in TBST for 1 hour at room temperature. They were then incubated overnight at 4 °C with primary antibodies, followed by incubation with HRP-conjugated anti-mouse or anti-rabbit secondary antibodies (Bioworld, USA). Immunoreactive bands were visualized using the SuperSignal West Dura Extended Duration Substrate (Thermo Fisher Scientific, USA).

The primary antibodies were diluted as follows: anti-Flag (Sigma, F7425, 1:3000); anti-Myc (Abmart, M20002H, 1:3000); anti-HA (Abcam, ab9110, 1:2000); anti-*β*-actin (Proteintech, 66009-1-Ig, 1:2000); anti-tubulin (Proteintech, 66031-1-Ig, 1:3000); anti-VSV-G (Santa Cruz, sc-66180, 1:1000); anti-YOD1 (Thermo, PA5-89126, 1:1000); and anti-Nef (Abcam, ab63919, 1:1000).

### 2.5 Mass spectrometry

HEK293T cells were transfected with either a Flag-Nef plasmid or an empty vector. Following a 48-hour incubation period post-transfection, the cells were lysed using NP-40 buffer. Anti-Flag M2 affinity gel (A2220; Sigma-Aldrich, USA) was used to immunoprecipitate Flag-Nef and its interacting proteins from whole-cell lysates. The proteins were subsequently resolved via SDS-PAGE and visualized by staining with Coomassie Brilliant Blue. Protein bands corresponding to both the control and experimental conditions were excised and subjected to in-gel tryptic digestion. The resulting tryptic peptides were desalted using C18 ZipTips (Millipore) and analyzed with an Orbitrap Elite hybrid mass spectrometer (Thermo Fisher Scientific, USA) interfaced with a Dionex LC system. The spectral data were interrogated against the UniProt human protein database using Proteome Discoverer software (version 1.4; Thermo Fisher Scientific).

### 2.6 Proteomics analysis

Peripheral blood samples were collected from 16 HIV-infected individuals on suppressive antiretroviral therapy, stratified into immunological non-responders (INR, CD4^+^ T-cell count <200 cells/μL, n=8) and immunological responders (IR, CD4^+^ T-cell count ≥200 cells/μL, n=8). For proteomic profiling, serum samples were sent to BGI Genomics (Shenzhen, China), where protein extraction, BCA quantification, tryptic digestion, and nano-liquid chromatography-tandem mass spectrometry (LC-MS/MS) analysis were performed following the company’s standard operational workflows. Raw MS data were processed by BGI using MaxQuant software (version 2.0.1.0) searched against the UniProt human protein database, enabling protein identification and label-free quantification (LFQ). Upon receipt of the quantitative results from BGI, differentially expressed proteins (DEPs) between the INR and IR groups were defined using a fold-change threshold of >1.5 and a P-value <0.05 (Student’s t-test). Principal component analysis (PCA) and hierarchical clustering were applied to assess overall sample separation and expression patterns. Functional annotation of DEPs was conducted using Gene Ontology (GO) enrichment analysis for biological processes, cellular components, and molecular functions, as well as Kyoto Encyclopedia of Genes and Genomes (KEGG) pathway enrichment analysis. Additionally, Gene Set Enrichment Analysis (GSEA) was performed using the MSigDB database to identify pathways significantly enriched in the INR group, with significance set at a false discovery rate (FDR) <0.25. All bioinformatic and statistical analyses were performed using R software (version 4.1.0) and GraphPad Prism 8.0, with statistical significance set at P <0.05.

### 2.7 Immunofluorescence microscopy

HEK293T cells were co-transfected with plasmids expressing Myc-His-YOD1 and Flag-Nef. After washing with 1× PBS, cells were fixed with 4% paraformaldehyde on ice, permeabilized with 0.1% Triton X-100, and blocked with 3% bovine serum albumin (BSA). Cells were incubated with primary antibodies against Myc and Flag epitopes diluted in 1% BSA. After three washes with 1× PBS, cells were incubated with Alexa Fluor 488-conjugated goat anti-mouse IgG (A11001) or Alexa Fluor 594-conjugated goat anti-rabbit IgG (A11012). Cell nuclei were counterstained with DAPI (4′,6-diamidino-2-phenylindole). Fluorescence images were acquired using a Nikon A1 laser-scanning confocal microscope.

### 2.8 *In vitro* binding assay

To validate the direct physical interaction between YOD1 and Nef in a cell-free system, HEK293T cells were transfected with Myc-His-YOD1 expression plasmid or empty vector. At 48 h post-transfection, cells were harvested and lysed to obtain whole-cell lysates containing Myc-His-YOD1 or control lysates. Meanwhile, Flag-tagged Nef protein was expressed in HEK293T cells and purified using anti-Flag M2 affinity agarose beads (Sigma-Aldrich). The purified Flag-Nef protein was then incubated with equal amounts of whole-cell lysates expressing Myc-His-YOD1 or the empty vector control at 4 °C for 2 h with gentle rotation. Following incubation, immunoprecipitation was performed by adding anti-Flag M2 beads to the reaction mixtures, followed by overnight incubation at 4 °C. The immunoprecipitated complexes were washed thoroughly with lysis buffer, eluted with SDS-loading buffer, and subjected to SDS-PAGE. The presence of Flag-Nef and Myc-His-YOD1 in the precipitates was detected by Western blotting using anti-Flag and anti-Myc antibodies, respectively. This reciprocal immunoprecipitation approach was employed to confirm the specificity of the YOD1-Nef interaction *in vitro*.

### 2.9 HIV-1 virus stock preparation and infection

HEK293T cells, cultured in 10 cm dishes, were transfected with 30 μg of the HIV NL4–3 plasmid to create HIV-1 viral particles capable of replication. Forty-eight hours post-transfection, supernatants were collected and clarified by centrifugation at 3,500 rpm for 10 minutes. The clarified supernatants were then filtered through a 0.45 μm membrane and concentrated via ultracentrifugation at 30,000 rpm for 1.5 hours at 4 °C. The concentrated HIV-1 viral stock was aliquoted and stored at -80 °C. THP-1 cells, activated with PMA (80 ng/mL), or PBMCs were infected with HIV NL4–3 at a multiplicity of infection (MOI) of 0.5 or 1.0 in the presence of Polybrene (8 μg/mL) for 6 hours at 37 °C, with intermittent mixing. Subsequently, the cells were washed twice with 1× PBS. PBMCs were maintained in a complete medium supplemented with recombinant human IL-2 at a concentration of 20 U/mL until collection. Viral production was assessed by collecting culture supernatants from infected cells and performing a p24 antigen capture ELISA.

### 2.10 Patient samples, data, and ethics

This study included samples from HIV-1–infected patients who received antiretroviral therapy at the Fifth People’s Hospital of Suzhou between Jan.2022 and Dec.2023. 45 patients who achieved successful immune reconstitution and 72 patients with incomplete immune reconstitution were selected after two years of antiretroviral therapy. Incomplete immune reconstitution was defined as a CD4^+^ T-cell count below 200 cells/μL. The Ficoll method was used to isolate PBMCs from peripheral blood samples collected before the commencement of antiretroviral therapy. Written informed consent was obtained from all participants. The study protocol was approved by the Ethics Committee of the Fifth People’s Hospital of Suzhou (approval NO. K-2024-005-01).

### 2.11 Cycloheximide pulse-chase assay

The stability of the Flag-Nef protein was assessed using a cycloheximide (CHX) pulse-chase assay. In brief, HEK293T cells were co-transfected with plasmids expressing Flag-Nef and Myc-YOD1. Twenty-four hours after transfection, cells were treated with DMSO or cycloheximide (100 μg/mL), harvested at the indicated time points, and subjected to Western blot analysis. Data from three independent experiments were analyzed using ImageJ software and are presented as the mean ± standard deviation (SD).

### 2.12 Statistical analysis

Statistical significance between groups was assessed using two-tailed Student’s *t*-tests. *P*-values of < 0.05, < 0.01, and < 0.001 were considered statistically significant. NS indicates no significance. All statistical analyses were performed using GraphPad Prism software (version 8.3.0).

## 3 Results

### 3.1 Proteomic characteristics and identification of YOD1 in immunonon-responsive individuals

To elucidate the molecular mechanisms underlying discordant immune recovery, we performed comparative proteomic profiling of INR (n=8) and IR (n=8) samples. PCA showed clear cohort separation (PC1: 83.4%; PC2: 7.6%; **Figure 1A**). A total of 1834 differentially expressed proteins (DEPs) were identified (FC>1.5, P<0.05), with 1404 upregulated and 430 downregulated in the INR group (**Figure 1B**). Consistent cohort separation was observed in the expanded IR-vs-INR analysis (**Supplementary Figure 1A**).

**Figure 1:**
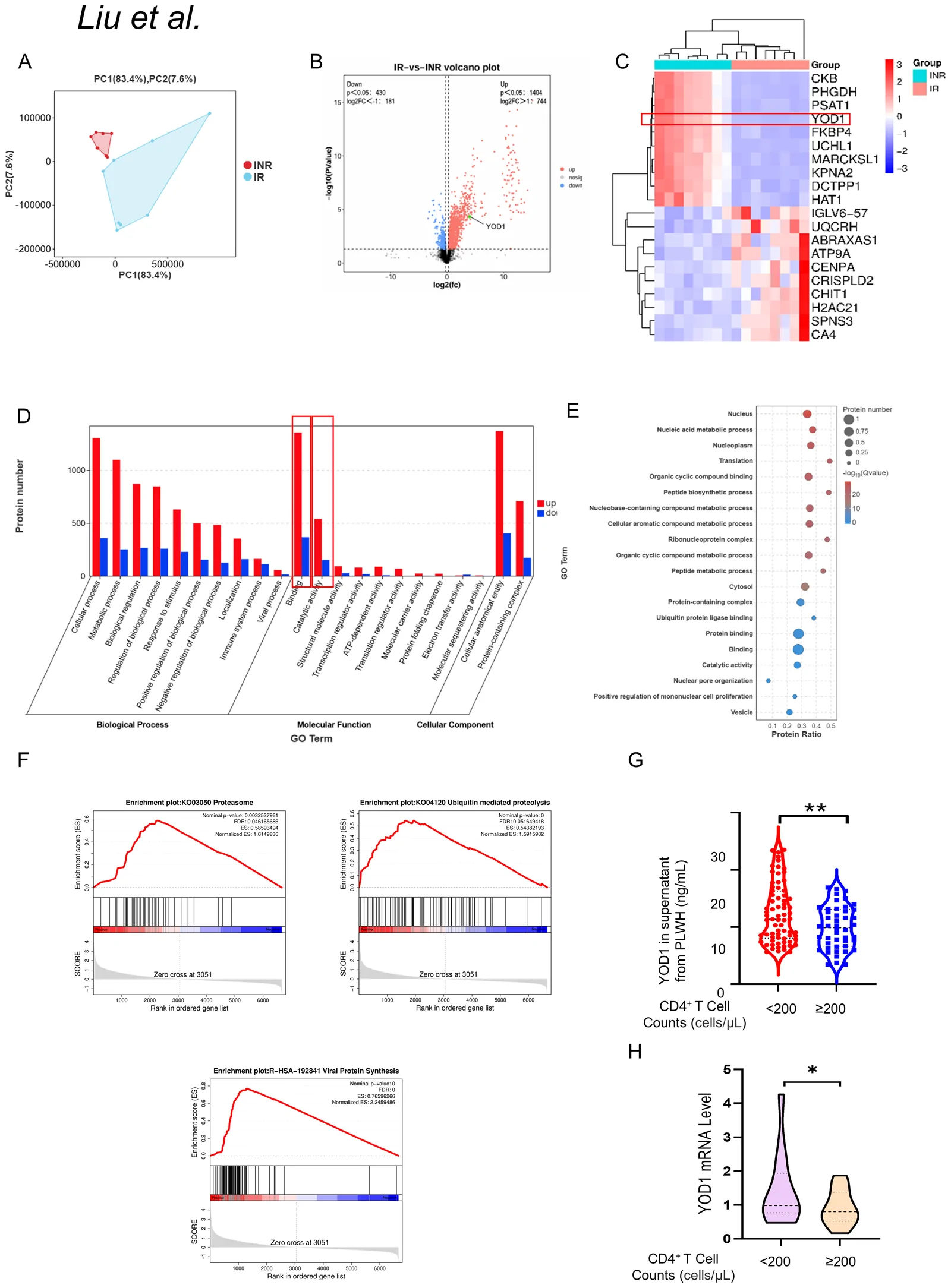
Proteomic characteristics and identification of YOD1 in immunonon-responsive individuals. **(A)** Comparative proteomic profiling was performed on samples from immunological non-responders (INR, n=8) and immunological responders (IR, n=8). Principal component analysis (PCA) shows the overall distribution of expression profiles across samples. **(B)** Volcano plot showing the distribution of differentially expressed proteins (DEPs). Using a fold change > 1.5 and P-value < 0.05 as filtering criteria, a total of 1,834 DEPs were identified, including 1,404 upregulated proteins (red) and 430 downregulated proteins (blue). **(C)** Clustering heatmap reveals distinct protein expression profiles between INR and IR groups, clearly distinguishing the two immune response states. **(D)** GO enrichment analysis of DEPs is presented as a bar plot showing significantly enriched GO terms (including biological process, BP; cellular component, CC; and molecular function, MF). **(E)** Differential GO enrichment analysis is displayed as a bubble plot illustrating significantly enriched signaling pathways. **(F)** Gene Set Enrichment Analysis (GSEA) reveals specific gene sets significantly enriched in the INR group. **(G)** EDTA-anticoagulated peripheral blood of AIDS patients was collected and serum was isolated. The level of YOD1 in plasma was detected by ELISA, and its correlation with CD4^+^ T cell counts was analyzed. **(H)** Peripheral blood samples were collected from AIDS patients and PBMCs were isolated. YOD1 mRNA expression level was detected by RT-qPCR after RNA extraction, and its correlation with CD4^+^ T cell counts was analyzed.

Hierarchical clustering confirmed distinct proteomic profiles, highlighting the deubiquitinase YOD1 as one of the most significantly upregulated proteins in INR samples, alongside PSAT1, CK8, and PHGDH (**Figure 1C**). GO analysis revealed that the DEPs were predominantly enriched in cellular and metabolic processes and molecular functions involving binding and catalytic activity (**Figures 1D, E**). The pronounced upregulation of catalytic activity proteins (red box in **Figure 1D**) was strongly linked to the ubiquitin-proteasome system. Correspondingly, KEGG pathway annotation (**Supplementary Figure 1B**) showed marked enrichment in pathways related to drug resistance, viral infection, and immune signaling (e.g., NOD-like receptor and TNF signaling).

GSEA demonstrated significant positive enrichment in the INR group for the proteasome (KO03050), ubiquitin-mediated proteolysis (KO04120), and viral protein synthesis (R-HSA-192841) pathways (**Figure 1F**). Supplementary GSEA further confirmed enhanced gene sets related to active ubiquitin synthesis and ubiquitin-dependent degradation (e.g., CDK2), alongside disruption of p53 signaling and cell cycle regulation (**Supplementary Figure 1C**). Collectively, these data suggest hyperactivated protein degradation machinery and dysregulated host immune/cell cycle networks in patients with poor immune recovery.

Given the prominence of the ubiquitin-proteasome axis, we focused on YOD1. Clinical validation confirmed that INR patients (CD4+ T cells <200 cells/µL) exhibited significantly higher serum YOD1 protein (**Figure 1G**) and mRNA levels (**Figure 1H**) compared to IR patients. These findings position YOD1 as a key mediator in the proteomic perturbations of immune non-recovery, providing a strong basis for subsequent mechanistic studies.

### 3.2 YOD1 Associates with HIV-1 Nef and selectively enhances its expression

Given the potential role of Nef in perturbing immune recovery, we sought to identify specific deubiquitinases (DUBs) that physically target this viral protein. HEK293T cells expressing Flag-Nef were subjected to immunoprecipitation with anti-Flag M2 agarose, followed by LC-MS/MS analysis. This screening revealed a panel of putative Nef-interacting DUBs, including YOD1, USP25, STAMBP, and CYLD (**Figure 2A**). Subsequent co-transfection of Flag-Nef with individual DUB plasmids demonstrated that YOD1 exerted the most robust stabilizing effect on Flag-Nef protein levels (**Supplementary Figure 2A**). Integrating these screening outcomes, YOD1 was designated as a primary candidate DUB for Nef.

**Figure 2:**
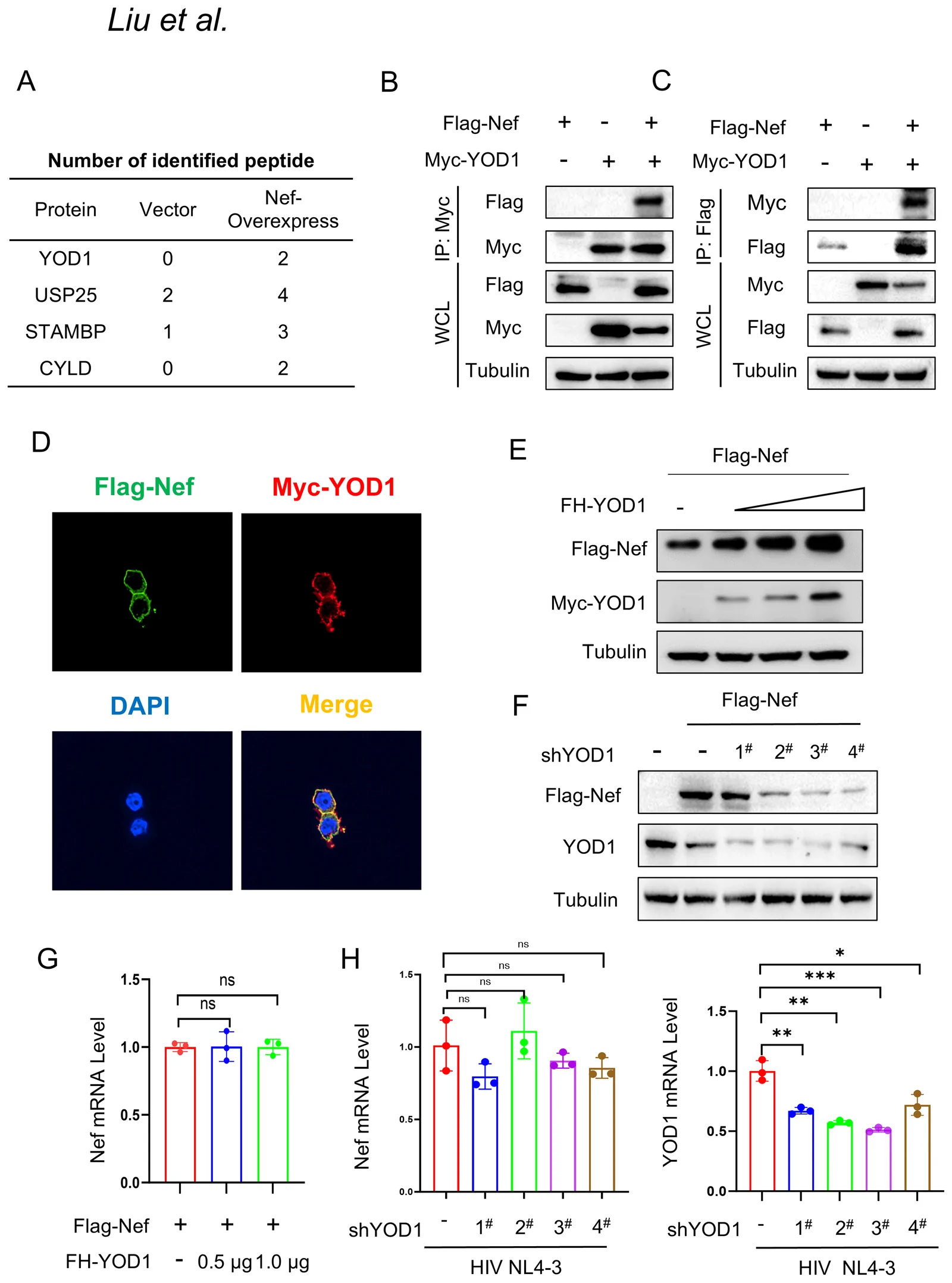
YOD1 associates with HIV-1 Nef and selectively enhances its expression. **(A)** HEK293T cells were transfected with either an empty vector or a Flag-Nef-expressing vector, followed by immunoprecipitation with FLAG affinity gels and mass spectrometry to identify potential deubiquitinating enzymes interacting with Flag-Nef. **(B)** Co-immunoprecipitation using a Myc antibody confirmed the interaction between YOD1 and Nef in HEK293T cells co-transfected with Flag-Nef and Myc-YOD1. **(C)** The interaction between Nef and YOD1 was further confirmed by immunoprecipitation with an anti-Flag antibody in co-transfected HEK293T cells. **(D)** Immunofluorescence analysis showed co-localization of Myc-YOD1 and Flag-Nef in Hela cells. **(E)** HEK293T cells were transfected with Flag-Nef plasmids, in conjunction with either empty vectors (-) or varying concentrations of FH-YOD1 plasmids. Whole-cell lysates were subsequently subjected to immunoblot analysis using the specified antibodies. **(F)** 293T cells were co-transfected with vectors to express Flag-Nef alongside either control shRNAs (-) or four distinct shRNAs targeting YOD1 (shYOD1 1^#^-4^#^). Seventy-two hours post-transfection, whole cell lysates were analyzed via immunoblotting as described. **(G)** The relative mRNA expression levels of Nef were quantified using RT-qPCR in HEK293T cells co-transfected with vectors to express Flag-Nef and either empty vectors (-) or increasing amounts of Myc-YOD1 plasmids. **(H)** HEK293T cells were co-transfected with HIV NL4-3; vector and control shRNA **(-)** or YOD1 shRNAs (shYOD1 1^#^-4^#^). Relative mRNA levels of HIV-encoded Nef and endogenous YOD1 were analyzed by RT-qPCR. ns: not significant, *p < 0.05, **p < 0.01, ***p < 0.001.

The physical association was further validated through reciprocal co-immunoprecipitation. When Myc-YOD1 was co-expressed with Flag-Nef, immunoprecipitation using either anti-Myc or anti-Flag antibodies successfully pulled down the interacting partner (**Figures 2B, C**). In contrast, CYLD, which was identified in the mass spectrometry data, failed to co-precipitate with Nef (**Supplementary Figure 2B**). Additionally, immunofluorescence microscopy confirmed the intracellular co-localization of Myc-YOD1 with Flag-Nef (**Figure 2D**).

Following the confirmation of their interaction, we investigated the regulatory impact of YOD1 on Nef expression. Transfection of increasing amounts of FH-YOD1 resulted in a dose-dependent accumulation of Flag-Nef (**Figure 2E**). To assess target specificity, parallel experiments were conducted with other HIV-1-encoded proteins (gp120, Vif, and Tat); however, YOD1 overexpression failed to alter their steady-state levels (**Supplementary Figure 2C**). Conversely, silencing endogenous YOD1 using four distinct shRNAs (shYOD1^#^–4^#^) markedly reduced Nef expression (**Figure 2F**). To rule out transcriptional regulation, Nef mRNA levels were measured via RT-qPCR following both YOD1 overexpression and knockdown; no significant changes were observed in either condition (**Figures 2G, H**). Collectively, these results demonstrate that YOD1 physically engages with Nef and positively governs its protein stability via post-translational mechanisms, independent of mRNA transcription.

### 3.3 YOD1 antagonizes Nef ubiquitination and degradation by targeting K6-linked ubiquitin chains

We further investigated the molecular mechanism underlying YOD1-mediated stabilization of the HIV-1 Nef protein. To determine whether YOD1 knockdown-induced Nef degradation occurs via the proteasome pathway, the proteasome inhibitor MG132 was employed. Treatment with MG132 prevented the reduction of HIV-1 Nef protein levels induced by siYOD1 (**Figure 3A**). These findings suggest that the HIV-1 Nef protein is degraded through the proteasome pathway.

**Figure 3:**
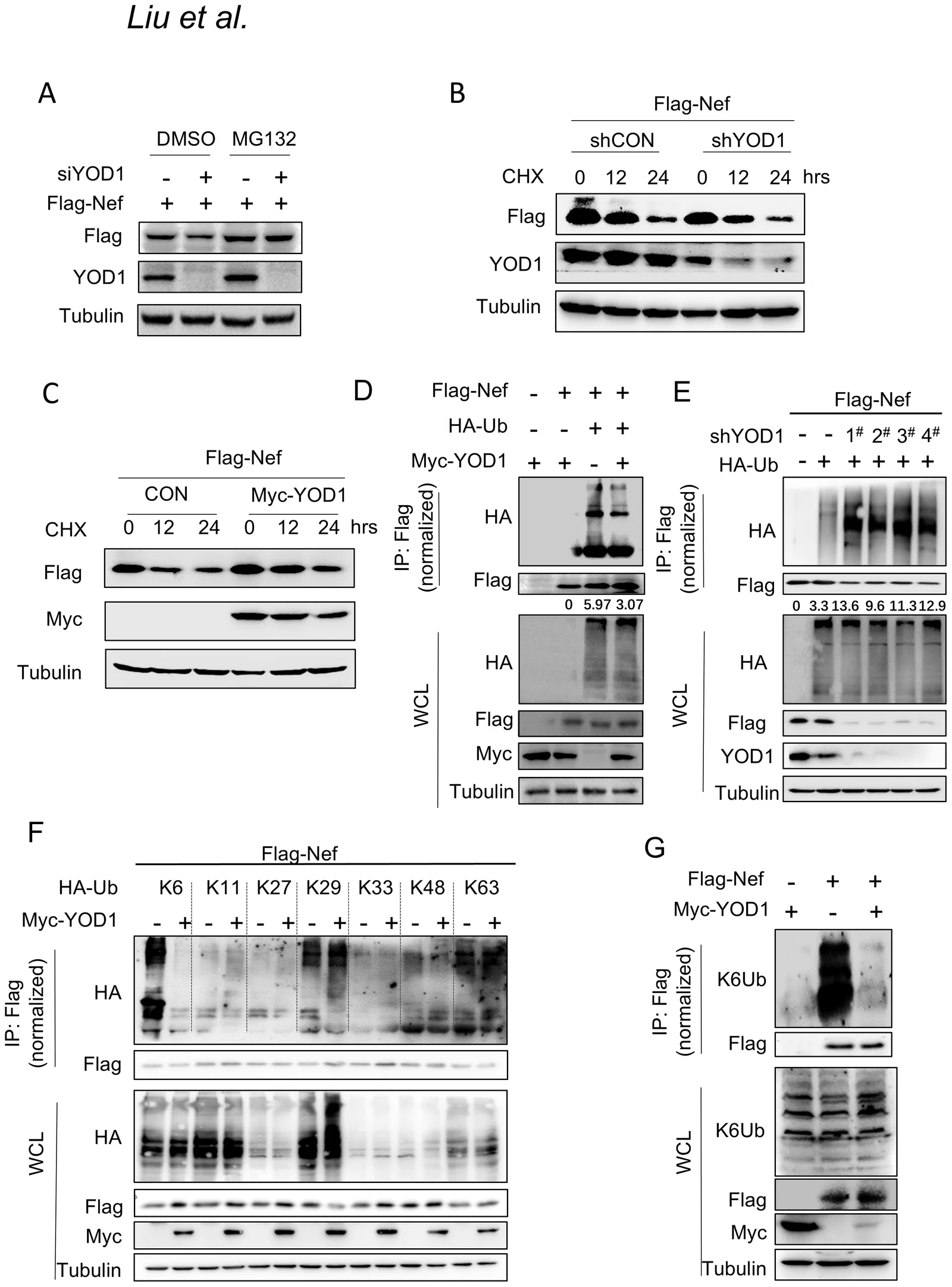
YOD1 antagonizes Nef ubiquitination and degradation by targeting K6-linked ubiquitin chains. **(A)** HEK293T cells were transfected with Flag-Nef and/or siYOD1 plasmids, treated with 10 µM MG132 or left untreated for 12 hours, and then analyzed for Nef protein levels via immunoblotting. **(B)** HEK293T cells were transfected with control shRNAs or shYOD1 along with Flag-Nef, then treated with cycloheximide (CHX) for 0, 12, and 24 hours, and analyzed by immunoblotting. **(C)** HEK293T cells were transfected with empty vectors or Myc-YOD1 and Flag-Nef, treated with CHX for 0, 12, and 24 hours, and analyzed by immunoblotting. **(D)** HEK293T cells were transfected with empty vectors or Myc-YOD1, along with Flag-Nef and HA-Ub, and Flag-Nef was immunoprecipitated using FLAG beads to assess its ubiquitination by immunoblotting. **(E)** HEK293T cells were transfected with control shRNAs or shYOD1 (1^#^-4^#^) plasmids, together with Flag-Nef and HA-Ub plasmids as indicated. Flag-Nef proteins were immunoprecipitated using FLAG beads to assess its ubiquitination by immunoblotting. **(F)** HEK293T cells were transfected with Flag-Nef and/or Myc-YOD1 plasmids, together with HA-Ub-K6 (R6K), K11(R11K), K27(R27K), K29(R29K), K33(R33K), K48(R48K), K63(R63K) plasmids. Flag-Nef proteins were immunoprecipitated by anti-Flag M2 agarose, and then Flag-Nef ubiquitination levels were analyzed by immunoblotting. **(G)** HEK293T cells transfected with Flag-Nef and/or Myc-YOD1 plasmids, Flag-Nef proteins were immunoprecipitated by anti-Flag M2 agarose, and then the levels of K6-linked ubiquitination of Flag-Nef were analyzed as indicated.

As YOD1-mediated upregulation of Nef expression did not occur through increased Nef transcription, we hypothesized that YOD1 participates in post-translational modification of Nef. We next evaluated the stability of the HIV-1 Nef protein using a cycloheximide (CHX) pulse-chase assay, in which CHX inhibits protein biosynthesis. HEK293T cells were transfected with shCON or shYOD1, and Nef protein stability was markedly reduced in YOD1-knockdown cells compared with controls (**Figure 3B**). Furthermore, overexpression of YOD1 noticeably inhibited Nef protein degradation (**Figure 3C**). These findings reveal that YOD1 enhances HIV-1 Nef protein expression by increasing its stability.

It is well established that deubiquitinating enzymes (DUBs) regulate substrate protein levels by removing ubiquitin chains. As YOD1 belongs to the ovarian tumor (OTU) family of DUBs, we hypothesized that it may regulate Nef ubiquitination. To test this hypothesis, Flag-Nef, HA-Ub, and Myc-YOD1 plasmids were co-transfected into HEK293T cells as designed. After 48 h, Flag-Nef was immunoprecipitated using anti-Flag M2 agarose, and ubiquitination levels were analyzed by Western blotting. Overexpression of YOD1 significantly reduced Nef ubiquitination (**Figure 3D**). We next analyzed Nef ubiquitination following YOD1 knockdown. HEK293T cells were co-transfected with Flag-Nef and HA-Ub and either control shRNAs (-) or four shYOD1 (1^#^-4^#^) plasmids, and Flag-Nef was immunoprecipitated using anti-Flag M2 agarose after 72 h. YOD1 knockdown astonishingly enhanced ubiquitination of the Flag-Nef protein (**Figure 3E**). These findings indicate that YOD1 stabilizes Nef by inhibiting its ubiquitination.

We then sought to determine which ubiquitin linkage specific type of YOD1 removes from Nef. HEK293T cells were co-transfected with Myc-His-YOD1, Flag-Nef plasmids, together with HA-Ub-K6 only (K6), HA-Ub-K11 only (K11), HA-Ub-K27only (K27), HA-Ub-K29 only (K29), HA-Ub-K33 only (K33), HA-Ub-K48 only (K48) and HA-Ub-K63 only (K63) plasmids. The result revealed that overexpression of YOD1 specifically reduced the formation of K6-linked polyubiquitin chains on Nef, and finally leading to the increased stability of Nef protein (**Figure 3F**). Subsequent transfection experiments further validated that YOD1 downregulated K6-linked polyubiquitination of Nef and stabilized its protein levels, thus ultimately contributing to the stabilization of its expression (**Figure 3G**). Taken together, these data indicate that cellular YOD1 acts as a deubiquitinating enzyme to cleave K6-linked ubiquitin chains from the HIV-1 Nef protein, thereby preventing its proteasomal degradation. However, this phenomenon may reflect a non-physiological state. Therefore, further experiments involving YOD1 silencing are needed to assess Nef protein stability.

### 3.4 YOD1 positively regulates Nef expression in the context of HIV-1 infection

Having established the YOD1-mediated regulation of Nef ubiquitination *in vitro*, we extended our investigation to clinical cohorts and viral infection models. Immunoprecipitation of Nef from PBMCs of people living with HIV (PLWH) revealed robust polyubiquitination of the endogenous viral protein (**Figure 4A**). Furthermore, reciprocal co-IP assays on the same clinical lysates demonstrated an interaction between Nef and YOD1 (**Figure 4B**). Consistent with these findings, the physical engagement between Nef and YOD1 was also confirmed in HIV-1-infected HEK293T cells and through *in vitro* binding assays (**Supplementary Figures 3A, B**).

**Figure 4:**
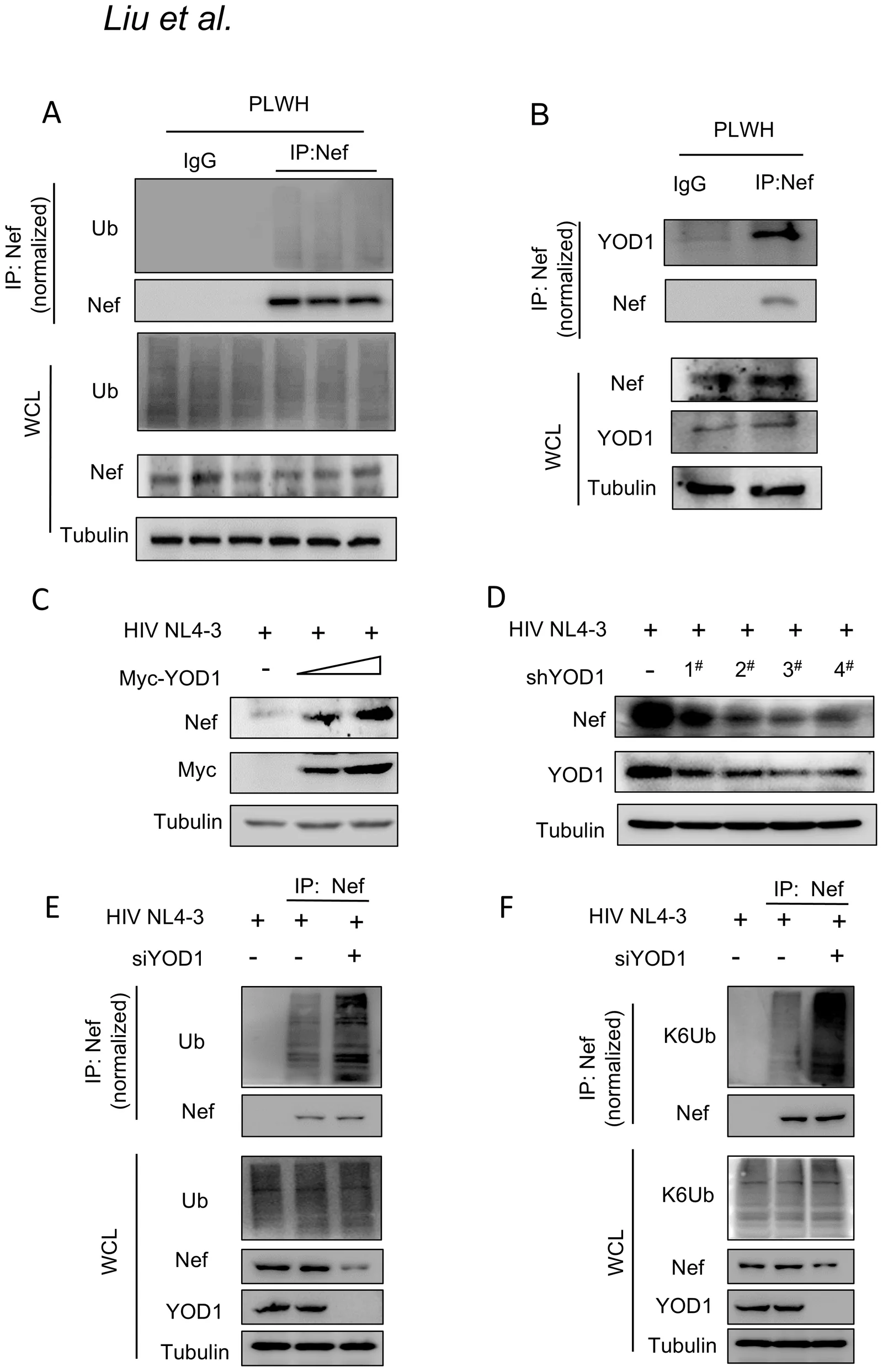
YOD1 positively regulates Nef expression in the context of HIV-1 infection. **(A)** Peripheral blood mononuclear cells (PBMCs) from people living with HIV-1 (PLWH) were lysed and subjected to immunoprecipitation with an anti-Nef antibody (IP: Nef) or IgG as a negative control. The immunoprecipitates were analyzed by Western blotting to detect endogenous Nef ubiquitination. **(B)** In PBMCs from people living with HIV-1 (PLWH), immunoprecipitation was performed with IgG or anti-Nef antibody (IP: Nef), followed by Western blotting for YOD1. **(C)** HEK293T cells were co-transfected with HIV NL4–3 molecular clone and Myc-YOD1 plasmids, and Nef protein levels were analyzed via immunoblotting. **(D)** HEK293T cells were co-transfected with HIV NL4–3 molecular clone and either control shRNAs or shYOD1 (1^#^-4^#^) plasmids, followed by immunoblotting of whole cell extracts. **(E)** THP-1 cells were transfected with siYOD1, infected with HIV NL4–3 for 24 h, and Nef ubiquitination was assessed by immunoprecipitation followed by Western blotting with anti-ubiquitin antibody. **(F)** Nef K6-linked ubiquitination was evaluated in siYOD1-transfected THP-1 cells after HIV-1 infection using a K6-linkage-specific ubiquitin antibody.

To evaluate the functional impact of this interaction on viral protein expression, we utilized the replication-competent HIV NL4–3 clone. Expression of YOD1 markedly enhanced the steady-state levels of virally produced Nef (**Figure 4C**). Conversely, knocking down YOD1 resulted in a substantial decrease in Nef expression within the infected cells (**Figure 4D**).

To dissect the underlying mechanism in a myeloid lineage, PMA-differentiated THP-1 cells were infected with HIV-1 following siRNA-mediated silencing of YOD1. Immunoprecipitation of Nef from these infected cells indicated that silencing YOD1 significantly intensified Nef polyubiquitination, specifically through the conjugation of K6-linked ubiquitin chains (**Figures 4E, F**). This observation was corroborated by targeted knockdown using distinct shRNAs (shYOD1 1^#^–4^#^), which similarly potentiated the ubiquitination of HIV-1-derived Nef (**Supplementary Figure 3C**). Collectively, these data indicate that endogenous YOD1 protects virally expressed Nef from K6-linked ubiquitination, consequently promoting its stability and enhancing viral protein accumulation during active infection.

### 3.5 The enzymatic activity of YOD1 is crucial for the stability of Nef

An exogenous expression experiment was conducted using the catalytically inactive mutant plasmid Myc-YOD1 (C/S). Consistent with the results shown in **Figures 2E**, wild-type YOD1 stabilized the Nef protein. Loss of catalytic activity in Myc-YOD1 abolished its ability to stabilize Nef (**Figure 5A**). Accordingly, the Nef protein produced by HIV-1 became unstable in the presence of the C155S mutant, whereas wild-type YOD1 stabilized the Nef protein expressed from HIV NL4-3 (**Figure 5B**).

**Figure 5:**
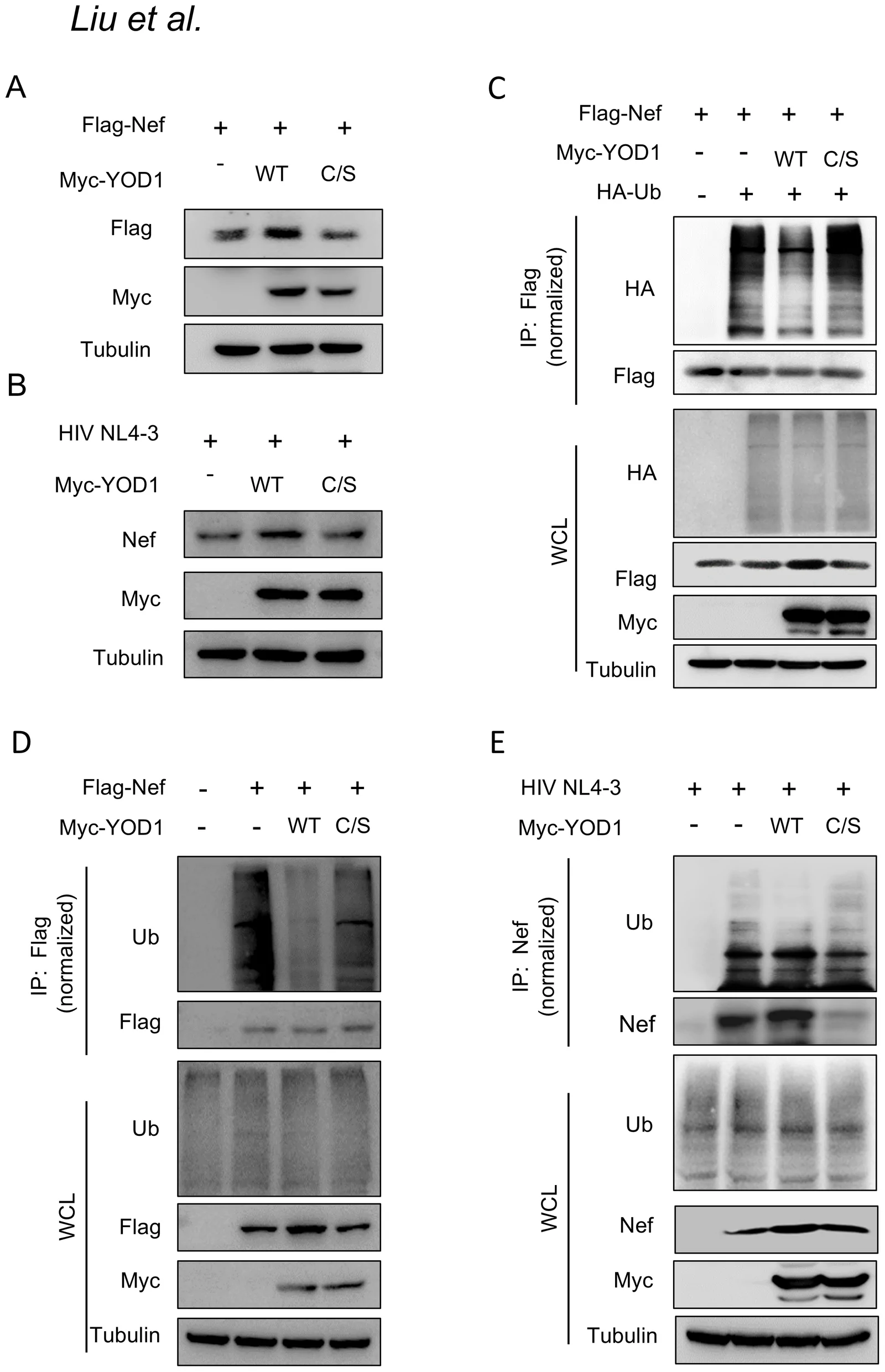
The enzymatic activity of YOD1 is crucial for the stability of Nef. **(A)** HEK293T cells were transfected with the vectors to express Flag-Nef and either Myc-YOD1 wild type (WT) or Myc-YOD1 C155S mutant. Nef protein levels were analyzed by immunoblotting as indicated. **(B)** HEK293T cells were transfected with an infectious HIV NL4-3, together with empty vectors (-) or Myc-YOD1 wild type (WT) or Myc-YOD1 C155S mutant plasmids, to produce HIV-1 virions. The protein levels of Nef produced by HIV NL4–3 virions were analyzed by immunoblotting as indicated. **(C)** HEK293T cells were transfected with empty vectors (CON) or Myc-YOD1 wild type (WT) or Myc-YOD1 C155S mutant plasmids, together with Flag-Nef and HA-Ub as indicated. Flag-Nef proteins were immunoprecipitated by Flag beads, and Flag-Nef ubiquitination levels were analyzed by immunoblotting. **(D)** HEK293T cells were transfected with empty vectors (CON) or Myc-YOD1 wild type (WT) or Myc-YOD1 C155S mutant plasmids, together with Flag-Nef as indicated. Flag-Nef proteins were immunoprecipitated by Flag beads, and Flag-Nef ubiquitination levels were analyzed by immunoblotting. **(E)** HEK293T cells were co-transfected with HIV NL4–3 molecular clone, together with empty vectors (−) or Myc-YOD1 wild type (WT) or Myc-YOD1 C155S mutant plasmids. Nef proteins were immunoprecipitated, and Nef ubiquitination levels were analyzed by immunoblotting.

As YOD1 was shown to reduce Nef ubiquitination, we next examined whether the catalytic mutant affected Nef ubiquitination levels. In HEK293T cells co-expressing Nef and HA-Ub, the C155S mutant failed to suppress Nef ubiquitination compared with wild-type Myc-YOD1 (**Figure 5C**). Moreover, intracellular expression of Myc-YOD1 (C155S) no longer reduced endogenous Nef ubiquitination (**Figure 5D**). Furthermore, YOD1 suppresses ubiquitination of HIV-1 NL4-3-derived Nef, but the catalytic mutant loses this deubiquitinating activity (**Figure 5E**).

These results demonstrate that YOD1 inhibits Nef degradation via its deubiquitinating activity, which is dependent on its catalytic cysteine residue. Thus, suppressing YOD1 expression during HIV-1 infection promotes Nef degradation, revealing a potential therapeutic strategy to limit viral replication.

### 3.6 YOD1 sustains HIV-1 Nef stability to promote viral infectivity

To investigate the interplay between YOD1 and Nef during active HIV-1 infection, PMA-differentiated THP-1 macrophages were inoculated with escalating doses of the HIV NL4–3 strain. A dose-dependent increase in both YOD1 and Nef protein levels was observed (**Figure 6A**). Moreover, the interaction between YOD1 and Nef exhibited a time-dependent pattern following viral infection (**Figure 6B**).

**Figure 6:**
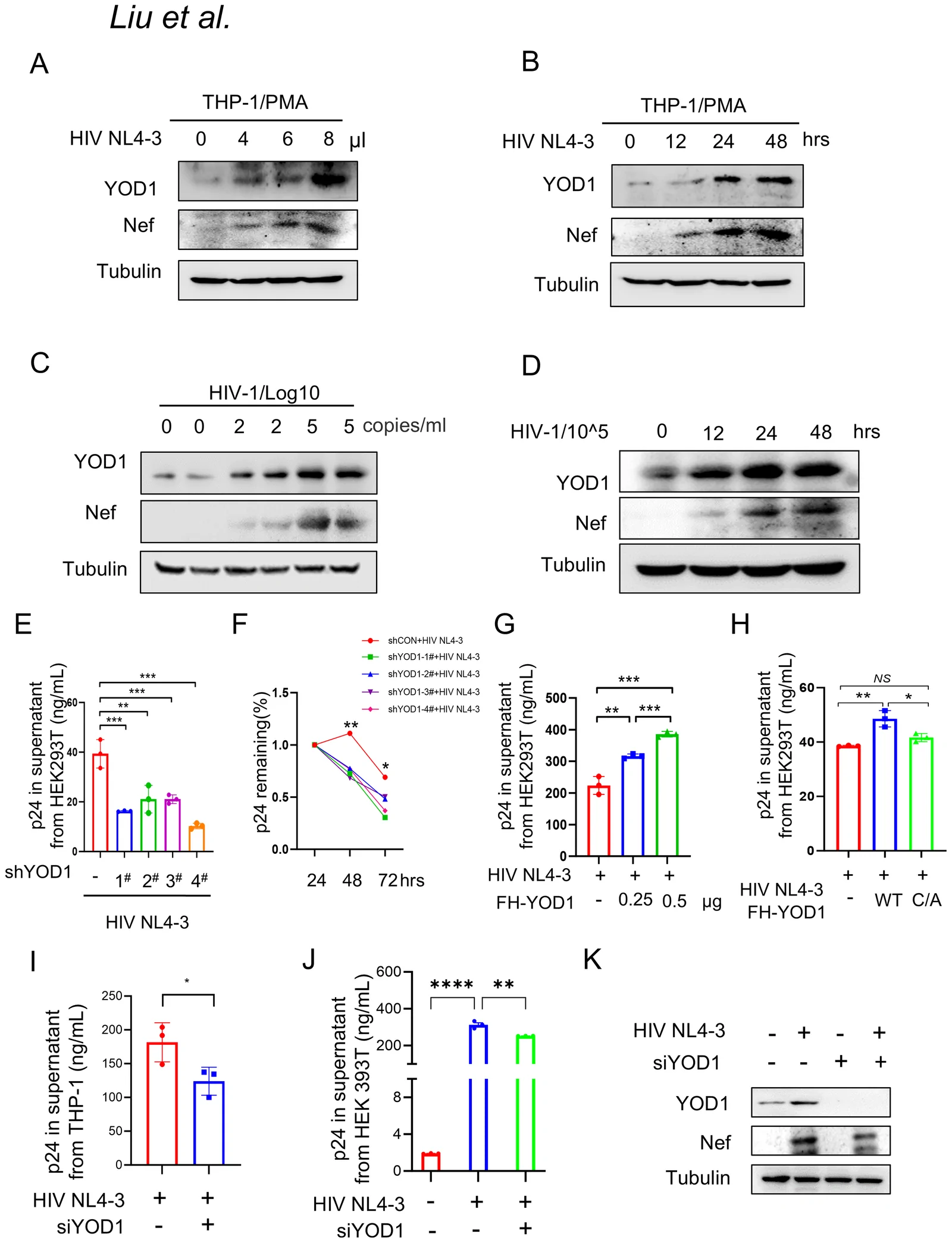
YOD1 sustains HIV-1 Nef stability to promote viral infectivity. **(A)** THP-1 cells activated by PMA (THP-1/PMA cells) were infected with HEK293T cell-derived HIV NL4–3 virus at different doses (0, 4, 6, and 8 μL). After 24 hours, cellular proteins were collected and YOD1 protein levels were detected by Western Blot. **(B)** THP-1/PMA cells were infected with HIV NL4–3 virus, and cellular proteins were collected at 0, 12, 24, and 48 hours after infection, and YOD1 protein levels were detected by Western Blot. **(C)** EDTA-anticoagulated peripheral blood of AIDS patients was collected, PBMC was separated by lymphocyte separation medium, and the protein expression of YOD1, Nef and Tubulin was detected by Western Blot. **(D)** THP-1/PMA cells were infected with the serum of AIDS patients with a viral load of 10^5^ copies/mL. Cellular proteins were collected at 0, 12, 24 and 48 hours after infection, and the protein expressions of YOD1, Nef and Tubulin were detected by Western Blot. **(E)** HEK293T cells were co-transfected with either control shRNAs or shYOD1 (1^#^-4^#^), together with HIV NL4-3, and the cell culture supernatant was collected 72 hours later. After centrifugation, the p24 antigen level in the supernatant was detected by ELISA. **(F)** HEK293T cells were transfected with plasmids as **(E)**, Cell supernatant was collected at 24, 48, and 72 hours after transfection, and HIV-1 p24 antigen was detected by ELISA. **(G)** HEK293T cells were co-transfected with gradient YOD1 and HIV NL4–3 plasmids, and the supernatant was collected 48 hours later to detect HIV-1 p24 antigen by ELISA. **(H)** HEK293T cells were transfected with empty vectors (CON) or FH-YOD1 WT/C155A mutants, and co-transfected with HIV NL4–3 for 48 h. Supernatants were collected and assayed for HIV-1 p24 antigen by ELISA. **(I, J)** THP-1/PMA cells/HEK293T cells were transfected with siYOD1, infected with HIV-1 NL4-3–48 hours later, and supernatants were collected 24 hours post-infection for p24 antigen measurement by ELISA. **(K)** HEK293T cells were similarly treated, and cell lysates were immunoblotted for YOD1 and Nef. NS, not significant; *P < 0.05; **P < 0.01; ***P < 0.001, ****P < 0.0001.

To validate these *in vitro* findings, PBMCs were isolated from PLWH stratified by viral loads (undetectable [set as 0], 10², and 10^5^ copies/mL) using EDTA-K2 anticoagulant tubes and Lymphocyte Separation Medium. Immunoblotting demonstrated a positive correlation between viral load and YOD1 expression. Consistently, YOD1 protein levels showed a positive correlation with Nef expression in patient-derived PBMCs (**Figure 6C**). Furthermore, both YOD1 and Nef levels increased with the duration of infection (**Figure 6D**). These results suggest that YOD1 may modulate host immune responses through Nef stabilization, particularly under conditions of high viral load and prolonged infection.

Given the role of YOD1 in stabilizing Nef, and the established attenuation of viral infectivity caused by Nef deficiency, we next assessed the impact of YOD1 on HIV-1 infectivity. HEK293T cells were co-transfected with the HIV NL4–3 proviral plasmid alongside shCON or shYOD1 constructs (1^#^–4^#^), and p24 antigen levels were measured in the culture supernatants. Knockdown of YOD1 significantly reduced p24 production (**Figure 6E**), with a notably faster decline in p24 levels over time compared to controls (**Figure 6F**). Conversely, YOD1 overexpression enhanced HIV-1 particle production in a dose-dependent manner (**Figure 6G**); however, the catalytically inactive FH-YOD1 (C155A) mutant failed to exert this effect (**Figure 6H**). Additionally, consistent results were obtained via ELISA in both HEK293T and THP-1 cells following siRNA-mediated silencing of endogenous YOD1 and subsequent HIV NL4–3 infection (**Figure 6I, J**). Western blotting further confirmed that loss of YOD1 significantly downregulated Nef expression, corroborating that host factor YOD1 is a critical regulator sustaining Nef stability in macrophage models as well (**Figure 6K**).

Therefore, YOD1 enhances HIV-1 pathogenicity during infection by stabilizing Nef, potentially contributing to impaired immune reconstitution and reduced efficacy of antiretroviral therapy in PLWH.

### 3.7 Vitamin C enhances anti-HIV-1 efficacy and promotes immune recovery by targeting YOD1 degradation

Co-IP and immunoblotting have established YOD1 as a critical host factor that enhances HIV-1 infectivity by stabilizing the Nef protein. Consequently, pharmacologically targeting YOD1 may serve as a viable strategy to bolster host antiviral defenses. Previous studies have indicated that essential nutrients, such as vitamins, can modulate host protein levels to fortify antiviral responses. Based on this, we screened a panel of vitamins (VA, VB, VC, VD, VE) for their ability to regulate YOD1 expression. Notably, Vitamin C (VC) significantly suppressed YOD1 expression, an effect consistently validated across both HEK293T and THP-1 cell lines (**Figure 7A**). In addition, CCK-8 assays confirmed that VC treatment did not compromise the viability of THP-1 or HEK293T cells, indicating no detectable cytotoxicity under these conditions. (**Figure 7B**).

**Figure 7:**
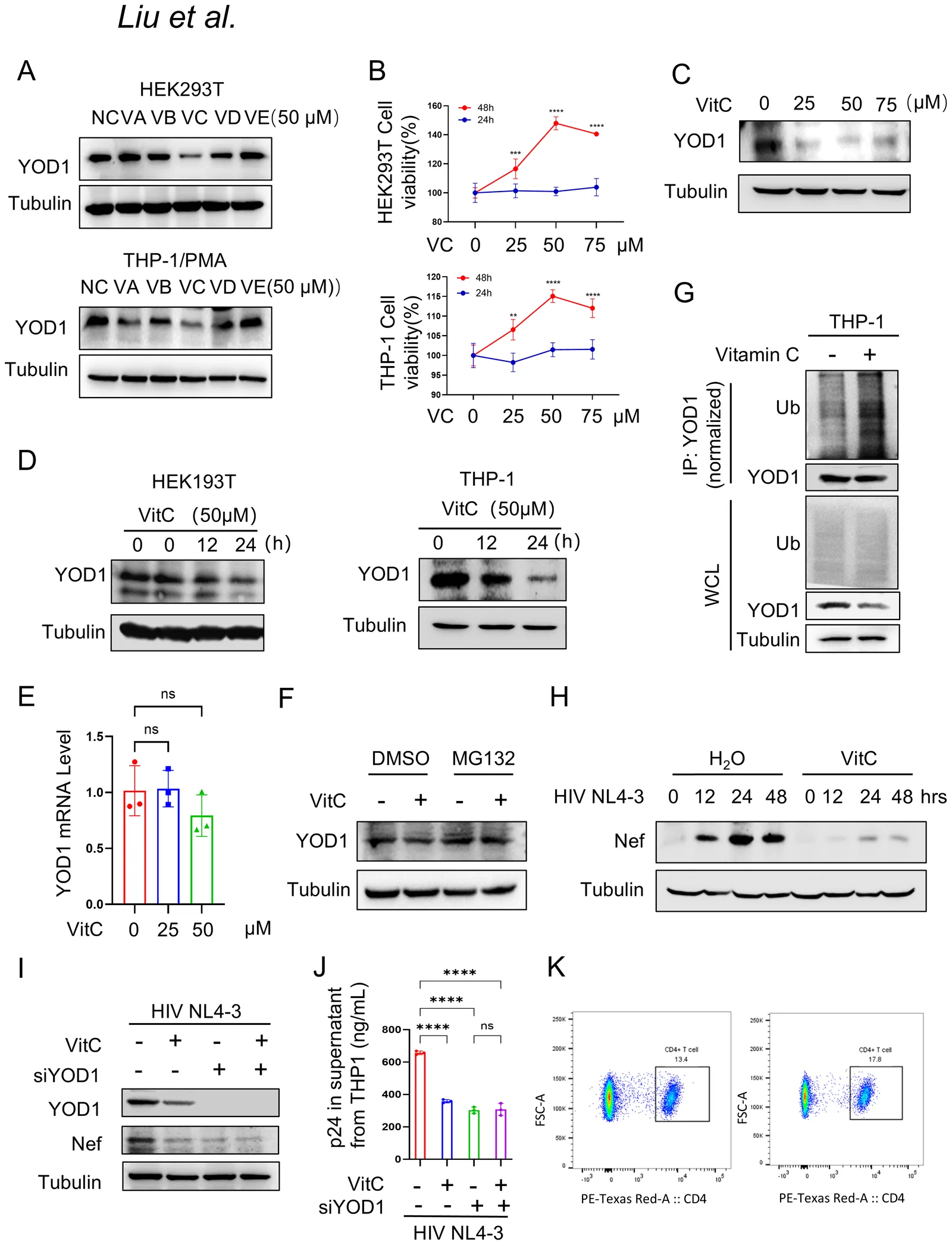
Vitamin C enhances anti-HIV-1 efficacy and promotes immune recovery by targeting YOD1 degradation. **(A)** HEK293T and THP-1/PMA cells were treated with different types of vitamins (Vitamin A, B, C, D, E; all at 50 µM) for 24 hours. Protein expression levels of YOD1 and Tubulin were examined by Western blotting. **(B)** HEK293T and THP-1 cells were treated with different concentrations of vitamin C (0, 25, 50, 75 µM) for 24 and 48 hours. Cell proliferation was assessed by CCK8 assay. **(C)** HEK293T cells were treated with different concentrations of vitamin C (0, 25, 50, 75 µM) for 48 hours. Protein expression levels of YOD1 and Tubulin were examined by Western blotting. **(D)** HEK293T cells and THP-1/PMA macrophages were treated with vitamin C (50 µM) for different time periods (0, 12, 24 hours). Protein expression levels of YOD1 and Tubulin were examined by Western blotting. **(E)** HEK293T cells were treated with different concentrations of vitamin C (0, 25, 50 µM) for 48 hours. YOD1 mRNA expression levels were measured by RT-qPCR. **(F)** THP-1 cells were treated with DMSO (control), MG132 (proteasome inhibitor, 10 µM), or vitamin C (50 µM). Protein expression levels of YOD1 and Tubulin were examined by Western blotting. **(G)** THP-1 cells were treated with vitamin C (50 µM) or control (H_2_O), followed by immunoprecipitation of YOD1 (IP: YOD1). YOD1 ubiquitination levels were detected using an anti-ubiquitin antibody (Ub). **(H)** THP-1/PMA cells were infected with HIV NL4–3 virus and treated with vitamin C (50 µM) or control. Cell lysates were collected at different time points post-infection (0, 12, 24, 48 hours). Protein expression levels of Nef, and Tubulin were examined by Western blotting. **(I)** HIV NL4-3-infected cells with or without YOD1 knockdown were treated with vitamin C or control, and the protein expression levels of YOD1 and Nef were verified by Western blotting. **(J)** In the presence of siYOD1 (or control siCON), HIV NL4-3-infected THP-1 cells were treated with vitamin C and the supernatant was collected 48 hours later to detect HIV-1 p24 antigen by ELISA. **(K)** CD4+ T cell count ratios were measured in HIV-1-infected cells treated with vitamin C.

Given that physiological plasma VC concentrations typically range from 50 to 70 μM, subsequent experiments were conducted using 50 μM VC. This treatment significantly downregulated YOD1 expression (**Figure 7C**). Time-course analysis revealed a gradual, time-dependent decrease in YOD1 protein levels following VC administration (**Figure 7D**). Mechanistically, RT-qPCR showed no significant change in YOD1 mRNA levels upon VC treatment (**Figure 7E**). However, the VC-induced degradation of YOD1 was effectively abrogated by the proteasome inhibitor MG132 (**Figure 7F**). Furthermore, VC treatment notably enhanced the polyubiquitination of YOD1 (**Figure 7G**). Treatment with the broad-spectrum DUB inhibitor PR-619 during HIV NL4–3 transfection significantly reduced both YOD1 and Nef protein levels (**Supplementary Figure 5A**), consistent with PR-619 promoting YOD1 degradation and consequently destabilizing Nef. Collectively, these data indicate that VC promotes YOD1 degradation at the post-translational level via the ubiquitin-proteasome pathway.

Next, we evaluated the functional consequences on viral proteins. Compared to controls, VC treatment significantly reduced Nef expression in HIV NL4-3-infected cells (**Figure 7H**). To determine whether this inhibitory effect was YOD1-dependent, rescue experiments were performed. As expected, the VC-mediated suppression of Nef was abolished upon YOD1 knockdown (**Figure 7I**). Consistent with this, ELISA showed that VC significantly suppressed the secretion of HIV-1 p24 antigen; however, this antiviral effect was entirely reversed in YOD1-deficient cells (**Figure 7J**). Finally, we assessed the impact of Vitamin C on immune recovery and observed that it markedly elevated CD4^+^ T-cell counts, confirming its ability to promote CD4^+^ T-cell reconstitution (**Figure 7K**).

In summary, Vitamin C exerts its anti-HIV-1 effects and facilitates CD4+ T cell recovery by promoting the ubiquitin-proteasome-mediated degradation of YOD1, thereby downregulating Nef expression. These findings offer a clinically accessible and potential adjuvant strategy for antiviral therapy and immune reconstitution in PLWH. However, the current experimental evidence regarding the relationship between vitamin C and the YOD1/Nef mechanism remains insufficient, and these conclusions require further validation and investigation in additional models and clinically relevant studies.

## 4 Discussion

The transition from early to late stages of HIV-1 infection is characterized by temporally regulated viral gene expression, with Tat, Rev, and Nef being among the earliest transcripts (28). Unlike other viral proteins that are confined to specific phases of the replication cycle, Nef persists throughout infection and distributes across multiple subcellular compartments, including the endoplasmic reticulum, plasma membrane, endosomes, and even exosomes. This unique spatiotemporal versatility enables Nef to function as a pleiotropic virulence factor that modulates receptor trafficking, signal transduction, and immune evasion. In this study, we began with a comparative proteomic profiling of immunological non-responders (INR) and immunological responders (IR) to identify host factors that might underpin the differential CD4^+^ T-cell recovery observed in these populations. This unbiased screening approach revealed that YOD1, a member of the ovarian tumor family of deubiquitinating enzymes, was among the most significantly upregulated proteins in INR patients, concomitant with global enrichment of ubiquitin-proteasome and viral protein synthesis pathways. This striking association prompted us to investigate whether YOD1 functionally intersects with HIV-1 pathogenesis. We subsequently uncovered a previously unappreciated layer of Nef regulation, whereby YOD1 removes K6-linked polyubiquitin chains and thereby shields Nef from proteasomal degradation. This post-translational control mechanism not only sustains Nef protein abundance but also enhances the infectivity of HIV-1 NL4-3 virions, providing a molecular rationale for the elevated Nef activity observed in immunologically non-responsive individuals. Consistently, our clinical data demonstrate that YOD1 expression correlates inversely with CD4^+^ T-cell recovery, suggesting that the YOD1-Nef axis may represent a previously unrecognized barrier to immune reconstitution.

Despite durable viral suppression achieved by contemporary antiretroviral therapy, a considerable fraction of HIV-infected individuals fail to restore CD4^+^ T-cell counts to normative levels, a condition termed immunological non-response that predisposes to both AIDS-defining and non-AIDS comorbidities, including malignancies, tuberculosis, opportunistic infections, and cardiovascular or cerebrovascular events (29). The mechanisms governing this incomplete recovery are multifactorial, involving persistent inflammation, microbial translocation, gut dysbiosis, co-infections, and metabolic disturbances (30). Our proteomic profiling adds a new dimension to this complexity by identifying YOD1 as one of the most significantly upregulated proteins in INR patients, alongside pronounced activation of ubiquitin-proteasome and viral protein synthesis pathways. This observation raises the possibility that deranged deubiquitinating activity may actively contribute to the maintenance of viral virulence factors, thereby perpetuating immune dysfunction even in the presence of effective virological control. The positive correlation between YOD1 levels and viral load in patient-derived PBMCs further supports this interpretation and positions YOD1 as a candidate biomarker for poor immunological outcomes.

Nef exerts its immunosuppressive effects partly through downregulation of CD4 surface expression via endocytic and lysosomal routes, a process that disrupts T-cell–antigen-presenting cell crosstalk, dampens T-cell activation, and facilitates efficient release of progeny virions (31, 32). Notably, ubiquitination of Nef at lysine 144 has been shown to be essential for this CD4-downmodulating function (33). Our findings extend this paradigm by demonstrating that YOD1-mediated deubiquitination not only preserves Nef stability but may also indirectly sustain its capacity to downregulate CD4, as YOD1 depletion led to reduced Nef expression and attenuated viral particle production. Although we did not directly measure CD4 surface levels in our experimental systems, the established linkage between Nef ubiquitination and CD4 internalization suggests that the YOD1-Nef axis could influence T-cell function beyond simple protein stabilization. Several small-molecule Nef inhibitors, including DLC27-14, B9, Compound 2C, and DQBS, have been developed to counteract Nef-mediated pathogenesis (34). Our identification of YOD1 as an upstream stabilizer of Nef offers an alternative intervention point—namely, pharmacological destabilization of Nef through YOD1 inhibition—which may synergize with Nef antagonists to achieve more complete suppression of Nef activity.

The ubiquitin–proteasome system (UPS) plays a central role in antiviral immunity by restricting viral replication. The nucleoproteins (NP) of influenza A virus (IAV) (35) and vesicular stomatitis virus (VSV) (36) undergo ubiquitin-mediated proteasomal degradation, thereby inhibiting viral infection. The SARS-CoV papain-like protease (PLpro) promotes viral replication by deubiquitinating TRAF6, thereby suppressing NF-κB activation (37). Ubiquitination also plays an essential regulatory role during the HIV-1 life cycle. Zhang et al. Zhang et al. (38) reported that the E3 ubiquitin ligase c-Cbl mediates K48-linked ubiquitination of Nef, promoting its proteasomal degradation and reducing Nef protein stability. The Gag polyprotein component p6 is monoubiquitinated at Lys27 and Lys33, which facilitates recruitment of TSG101 and promotes viral particle release (39). During HIV-1 infection, HIV integrase (IN) undergoes polyubiquitination and subsequent proteasomal degradation, which impairs proviral DNA formation and viral replication. The E3 ligase MDM2 mediates K63-linked polyubiquitination of Tat, facilitating recruitment of the cellular P-TEFb complex to viral promoters (40). Monoubiquitination of the HIV-1 accessory protein Vif, mediated by the E3 ligases NEDD4 and AIP4, promotes Gag recruitment to viral assembly and release sites (41). Conversely, the E3 ubiquitin ligases SCF^cyclin F and MDM2 promote proteasome-mediated degradation of Vif (42). Despite this wealth of knowledge, the landscape of DUBs acting on HIV-1 proteins has remained largely uncharted. Our identification of YOD1 as a Nef-directed DUB fills this gap and underscores the notion that the host DUB repertoire can be co-opted by viruses to fine-tune the stability of key pathogenic factors.

The deubiquitinating enzyme YOD1 belongs to the ovarian tumor (OTU) family, a highly conserved group involved in endoplasmic reticulum-associated degradation, clearance of damaged lysosomes, and immune-related cellular processes in mammals (43). Upon IL-1*β* stimulation, YOD1 dissociates from TRAF6, promoting TRAF6 ubiquitination and thereby inhibiting activation of the IKK/NF-κB signaling pathway (44). In antiviral immunity, YOD1 is recruited to mitochondria, where it removes K63-linked ubiquitin chains from MAVS, thereby preventing MAVS activation, disrupting antiviral signaling, and suppressing host immune responses (45). These prior observations collectively paint YOD1 as a negative modulator of host antiviral defenses. Our study adds a new facet to this immunoregulatory profile by demonstrating that YOD1 directly stabilizes a viral protein, Nef, thereby amplifying viral virulence. This dual role—suppressing host innate immunity while enhancing viral factor stability—positions YOD1 as a particularly attractive target for therapeutic intervention. Pharmacological inhibition of YOD1 could simultaneously relieve its suppression of MAVS-mediated interferon responses and promote Nef degradation, yielding a two-pronged antiviral effect.

The COVID-19 pandemic has highlighted the potential of nutritional modulators in antiviral strategies. SARS-CoV-2 exploits ACE2 for cellular entry, and Vitamin C has been shown to reduce ACE2 protein levels in a dose-dependent manner, thereby limiting viral uptake (46, 47). Interestingly, HIV-1 and SARS-CoV-2 share several host cellular factors and pathways involved in viral assembly, release, and envelope integration (48). HIV-1 infection is frequently accompanied by intestinal epithelial injury and CD4^+^ T-cell loss, and even with ART, mucosal damage recovers slowly. Altered ACE2 expression has been documented in the intestinal epithelium of HIV-positive individuals (49). Our findings reveal that Vitamin C downregulates both YOD1 and Nef protein levels, and that this effect is strictly YOD1-dependent, as YOD1 depletion abolished Vitamin C-mediated Nef suppression and antiviral activity. These observations extend the known antiviral repertoire of Vitamin C beyond ACE2 modulation to include destabilization of a HIV-1 virulence factor via YOD1 degradation. Whether this mechanism operates in intestinal tissues where both HIV and SARS-CoV-2 replicate warrants further exploration, but the convergence of Vitamin C action on two distinct viruses through related pathways is intriguing and may inform future adjuvant therapeutic strategies.

Collectively, this study provides the first evidence that YOD1 upregulates HIV-1 Nef protein levels by removing K6-linked ubiquitin chains, thereby preventing proteasomal degradation. Mutation of the catalytic cysteine residue (C155S) abrogates this activity and markedly reduces Nef-mediated viral virulence. Moreover, the positive correlation between YOD1 expression and impaired immune recovery suggests that YOD1 may serve as a predictive biomarker for CD4^+^ T-cell reconstitution in PLWH. Finally, the mechanistic connection we establish between Vitamin C and the YOD1-Nef regulatory axis offers a clinically accessible adjunctive strategy to enhance antiretroviral outcomes, particularly in the subset of patients who face persistent immune dysfunction despite virological suppression. Future work should focus on validating these findings in larger longitudinal cohorts and exploring the therapeutic window of YOD1-targeting interventions *in vivo*.

## Data Availability

The mass spectrometry proteomics data have been deposited to the ProteomeXchange Consortium vie the PRIDE partner repository with the dataset identifier PXD085805.
