# Supplementary material for: YOD1 stabilizes HIV-1 Nef via K6-linked deubiquitination to promote viral infectivity and impair immune recovery
Source: Front Immunol. 2026 Oct 23;17:1936616. doi: 10.3389/fimmu.2026.1936616 (PMC13739579; doi:10.3389/fimmu.2026.1936616)
Supplement: Supplementary file 1 [file Table1.docx]

**YOD1 Stabilizes HIV-1 Nef via K6-Linked Deubiquitination to Promote Viral Infectivity and Impair Immune Recovery**

**Jin Liu****^1#^, Qian Xu^2#^, Yu Jiang^3#^, Zhouzhou Zhang^4#^, Guiyan Li^1^, Minyan Yao^5^, Tiantian Gan^1^, Yiming Zheng^1^, Yujuan Yan^1^, Yueping Zhu^1^, Saihong Gu^1^, Ming Li^1^, Li Zhu^1^*, Jianping Zhang^5^*,** **Chuanwu Zhu^1^*, Feng Qian^1^*.**

^1^Department of Infectious Diseases, The Affiliated Infectious Diseases Hospital, Suzhou Medical College of Soochow University, The Fifth People's Hospital of Suzhou, Suzhou, China

^2^Department of Infectious Diseases, Zhangjiagang Hospital affiliated to Soochow University, Suzhou, China

^3^Department of Pharmacy, The Affiliated Infectious Diseases Hospital, Suzhou Medical College of Soochow University, The Fifth People's Hospital of Suzhou, Suzhou, China

^4^Department of Urology Surgery, The Affiliated Suzhou Hospital of Nanjing Medical University, Suzhou Municipal Hospital, Gusu School, Nanjing Medical University, Suzhou, China

^5^Department of Tuberculosis, The Affiliated Infectious Diseases Hospital, Suzhou Medical College of Soochow University, The Fifth People's Hospital of Suzhou, Suzhou, China

*Corresponding author:

Feng Qian, [fengqian70@126.com](mailto:fengqian70@126.com);

Chuanwu Zhu, zhuchw@126.com;

Jianping Zhang, zhangjianping_yb@suda.edu.cn;

Li Zhu, qixi_julie@163.com;

^#^These authors contributed equally to this work.

**Keywords:** **YOD1, HIV-1, Nef, Vitamin C, immune reconfiguration.**

Supplementary Data 1.

(A)Bar plot of differentially expressed genes (DEGs). The vertical axis represents the number of DEGs.

(B)KEGG pathway enrichment analysis of DEGs. The horizontal axis represents the number of genes enriched in each pathway, and the vertical axis represents the KEGG pathway names.

(C)Gene Set Enrichment Analysis (GSEA). The plot shows the enrichment of representative gene sets (including cell cycle, p53 signaling pathway, ubiquitin-mediated proteolysis) in the indicated comparison groups.

Supplementary Data 2.

(A)Flag-Nef and DUBs were transfected into HEK293T cells, identifying YOD1 as a ubiquitin-specific protease that stabilizes Nef protein levels.

(B) Co-immunoprecipitation using a Flag antibody confirmed the interaction between Flag-Nef and Myc-CYLD in HEK293T cells co-transfected with Flag-Nef and Myc-CYLD.

(C)HEK293T cells were transfected with either Flag-gp120, Flag-Tat, or Flag-Vif plasmids, in conjunction with either empty vectors (-) or varying concentrations of Myc-YOD1 plasmids. Whole cell lysates were subsequently subjected to immunoblot analysis using the specified antibodies.

Supplementary Data 3.

(A)HEK293T cells were transfected with HIV NL4-3 for 48 hours to produce virions. Immunoprecipitation was used to study the interaction between endogenous YOD1 and HIV Nef.

(B) Purified Flag‑tagged Nef was incubated with whole‑cell lysates expressing Myc‑YOD1 or empty vector. Immunoprecipitation (IP) was performed using anti‑Flag beads, and the precipitates were immunoblotted with anti‑Flag and anti‑Myc antibodies.

(C) HEK293T cells were co-transfected with HIV NL4-3 and either control shRNA or YOD1-specific shRNAs (shYOD1 1^#^-4^#^). Nef immunoprecipitation (IP: Nef) was performed, followed by immunoblotting for ubiquitinated Nef (Ub-Nef) using an anti-ubiquitin antibody.

Supplementary Data 4.

(A) Hela or HEK293T cells were co-transfected with Flag-Nef and Myc-His-YOD1. After immunostaining (DAPI, blue; Flag, green; Myc-His, red), confocal microscopy was performed. The merged images show significant co-localization of YOD1 (red) with Nef (green) in the cytoplasm (yellow signals), indicating spatial co-distribution of the two proteins.

Supplementary Data 5.

(A) THP-1/PMA cells and treated with DMSO or the deubiquitinase inhibitor PR-619 for 0, 12, 24, 36, and 48 hours. Whole-cell lysates were harvested and subjected to Western blot analysis to detect YOD1 and Nef protein levels.
